# *The Unsteady Mainstay of the Family*: Now Adult Children's Retrospective View on Social Support in Relation to Their Parent's Heart Transplantation

**DOI:** 10.1155/2014/541241

**Published:** 2014-11-06

**Authors:** Susanna Ågren, Bodil Ivarsson, Helén Rönning

**Affiliations:** ^1^Department of Medical and Health Sciences, Linköping University, 581 83 Linköping, Sweden; ^2^Department of Cardiothoracic Surgery, Linköping University, Linköping, Sweden; ^3^Department of Cardiothoracic Surgery, Clinical Sciences, Lund University, 221 85 Lund, Sweden; ^4^Department of Cardiothoracic Surgery/THAI, Skåne University Hospital and Medical Services, 221 85 Lund, Sweden; ^5^School of Health Sciences, Jönköping University, 551 11 Jönköping, Sweden

## Abstract

The needs for support among children with a seriously ill parent, who is waiting for heart transplantation, are unknown today. The aim was to describe now adult children's experiences of social support in relation to a parent's heart transplant during childhood. Nine females and four males were interviewed. The median age for the children was 18 at the transplantation and their parents had been ill before for 18 months (median) and on waiting list for 161 days (mean). Three categories emerged: *health care professionals' approaches, family and friends' approaches, and society approaches*. Our results show that there was lack of support for children of heart transplantation patients. Support in the shape of information was in most cases provided by the sick or healthy parent. It is of great clinical importance to develop psychosocial support programs for children with a seriously ill parent waiting for heart transplantation (before, during, and after surgery).

## 1. Introduction

According to Swedish law, health care professionals should pay particular attention to children in families where one parent has a serious physical illness [1]. Patients waiting for heart transplantation are living with a life-threatening disease, and between 1989 and 2011 a total of 822 heart transplants were performed in Sweden [2]. Even if the patients' condition is life-threatening, most of them are very hopeful about the future [3]. Patients who need a donor heart have described that the most stressful parts are knowing that their heart disease is incurable, that they need a heart transplant, and concerns for their family [4], particularly the children [5–7]. The situation of children with a parent seriously ill with a heart disease waiting for heart transplantation is unknown today.

For the patient waiting for heart transplantation, support from relatives is important [8]. It has been shown that relatives of patients waiting for heart transplantation experience an increased burden [9]. Patients waiting for heart transplantation with young children are concerned about their partners who have to take the main responsibility for their children's well-being on their own [10]. The Swedish Health Care law was strengthened in 2010 with regard to children with a parent with a serious physical illness. Health services have to pay particular attention to the children's need for information, advice, and support [1]. It has been shown that young carers with mentally unhealthy parents need support that includes knowledge and understanding. They need communication and shared experiences, advice and feedback, outside involvement, acute relief, and structured help with their care commitments [11].

Ireland and Pakenham [12] stated that children with a severely somatically ill parent worried more about the parent than children with a mentally ill parent. The risk for anxiety, depression, and somatic complaints is high in children with a chronically ill parent [13]. Thastum et al. [14] showed that children of patients with cancer are aware of the disease but that they are restricted by the emotional parent-child communication. The child observed both sick and healthy emotional states in the parent. The child's observations and expressions identified coping strategies used by younger people [14]. Patients wondered how their children were affected by the heart transplantation, but they had no forum where they could address these concerns [7]. If the family had a lower standard of living because of the parent's chronic illness, the children were affected too [13]. This indicates that children with an ill parent need social support. Social support is commonly categorized into emotional support, tangible/instrumental support, informational support, and appraisal support [15]. Emotional support refers to verbal and nonverbal communication, including love, empathy, encouragement, and comfort. Informational support refers to the provision of information and advice. Instrumental support involves concrete needs and aspects, such as financial support or support with household chores. Appraisal support occurs after constructive feedback and affirmation that someone can make an informed decision on their own. All four types of support are described to improve health outcomes [15]. The meaning of support may include encouraging someone to cope with life situations, helping them with the necessities of life, and being present [16]. However, to our knowledge there is a shortage of studies on children of patients who have undergone heart transplantation and their experiences of social support.

## 2. Aim

The aim was to describe now adult children's experiences of social support in relation to a parent's heart transplant during their childhood.

## 3. Methods

### 3.1. Design and Setting

The study had an explorative design, based on qualitative content analysis, with an inductive approach in order to provide knowledge and understanding of the phenomenon under study. The method was manifest content analysis [17]. To fulfill the aim, qualitative method of content analysis approach was chosen to analyze the children's experiences of social support in relation to a parent's heart transplant during childhood. The totality and context are important and trying to understand the unique of humans. The advantage of the qualitative content analysis is that it is adaptive; it can be used regardless of whether the material is abstracted and interpreted in a low or high level [18]. Qualitative content analysis can be applied to analyze experiences of an individual and focuses on the interpretation of audiotaped interview transcriptions [19]. Sweden has 9 million inhabitants and heart transplantation is performed in two hospitals. This study is a comprehensive survey of all children who have a parent (still in life) which has been transplanted in one of Sweden's two heart transplant centers. Now adult independent children in the age of 18 or older and able to read and understand Swedish language with experiences of a parent's heart transplant were recruited to participate in the study. All parents (twenty patients with children underwent heart transplantation at one of Sweden's two heart transplant centers) were contacted. Written and oral information were given to the parent, who further contacted their children. If the children wanted to participate in the study, the parent gives the children's name and contact details to the researchers. The now adult children had to be younger than 25 years of age during the heart transplantation. This is in accordance with the Youth Board in Sweden [20] which defines youth as <25 years of age and childhood can be seen as the period before that age. Of twenty parents, eight did not want their children to be interviewed. To collect data, telephone interviews were used as a way to bridge the geographical distance between the participants and interviewers [21]. Telephone interviews, one per participant, took place between May and December 2013 [22]. The analysis of the telephone interviews was conducted using a qualitative manifest content analysis as the data collected in the interviews did not allow interpretation of the underlying sentences [17].

### 3.2. Participants

Interviews were conducted with thirteen adult children who had a parent diagnosed with cardiomyopathy or congenital heart disease and had undergone heart transplantation. Of these participants, nine were females and four were males ranging from 23 to 37 years of age, with a median age of 26. Nearly 50% of the participants reported that their mothers had undergone heart transplantation. Two participants reported that their parents had got a divorce before the heart transplantation. Eight of thirteen participants reported that they lived at home permanently and the rest stayed at home from time to time during the period when the parent was seriously ill and at the time of the heart transplantation. The period from becoming sick to heart transplantation ranged from six months to ten years with a median time period of 18 months (time on waiting list mean 161 days, range 33 to 913 days). The median age of the children at the time of the heart transplantation was 18.

### 3.3. Data Collection and Analysis

In order to recruit participants the researchers contacted the adult children after the parents had forwarded their contact details. Oral information and written information (which had previously been sent by mail) were provided, and after agreeing to participation, oral and written informed consents were given. Date and time for the telephone interviews were then set. The interviews, one per participant, which were conducted as a dialogue, lasted between 15 to 30 minutes [22]. The opening question was what was your experience of support during your parent's heart transplantation? The participants were asked to talk more about the situation before, during, and after the parent's heart transplantation and their perceptions of support. All thirteen interviews were conducted by telephone and were audiotaped and transcribed verbatim. The transcribed interviews were analyzed using a qualitative content analysis based on [17]. First, all the researchers familiarized themselves with the interviews in order to obtain an overview of the content. Significant meaning units were identified, condensed, and coded. The codes were grouped and abstracted to categories and subcategories. First, two categories emerged:* health care professionals' approaches and the society approaches*. Independently of each other, all researchers critically examined the categories and reflected on the content which increased credibility [23]. The categories were refined and three categories now emerged:* health care professionals' approaches, the family and friends' approaches, *and the* society approaches*. Thereafter, all researchers discussed the content in the categories together in a critical and open dialogue, and in the final stage, all authors reached consensus regarding the content. Original direct quotations are present to illustrate the children's experiences.

### 3.4. Research Ethics

Participation in the study was voluntary and the participants could withdraw at any time without their care being affected. Informed consent was obtained through oral and written information where the voluntary nature of the study was emphasized. This gave the respondents an opportunity to reflect upon and independently evaluate possible participation in the study. Confidentiality was guaranteed. No dependence relationship existed between the participants and the researchers. The interviews provided an opportunity for the respondents to share their own experiences of support at the time of their parent's transplant. A potential ethical problem was if the interviews caused emotional reactions in the participants with regard to their experiences of the disease. The researchers were aware of these risks and as they have extensive experience of critically ill patients and research, this problem was minimized through sensitivity, consideration, and preparedness. A social worker was available to provide counselling to the participants. However, this was not needed. The study was approved by the participating clinical managers, and permission was granted by the Regional Ethical Review Board (Ref. number LU 638/2008).

## 4. Results

Three categories describing how support was provided before, during, and after the parent's heart transplantation emerged:* health care professionals' approaches, family and friends' approaches, and society approaches.*

### 4.1. Health Care Professionals' Approaches

None of the children felt that they received regular oral or written information or support by health care professionals during the period of illness. The children's feelings in connection with having a sick parent did not attract the attention of health care professionals.

*Then the doctors spoke with dad (the ill parent), and then he gave us further information… Perhaps it was decided that that's how it should be… (Child 6).*

The children experienced that they had been given some support and information in connection with the parent's hospitalization. The physicians who visited the patient briefly informed the children about some parts, and the nurses who cared for the patient spontaneously told them about, for instance, devices and intravenous catheters, which helped the children to understand the parent's situation.

*The nurses gave us a lot of information and support by showing and explaining when he was on the ventilator, and when he had a ventricular assist device as well. Before we went in they explained that there would be a lot of tubes and they stood next to us when we went in to see him, and that was probably good support (Child 7).*

The children wanted the health care professionals to involve them in the process and in what was going on by asking about their need for information and not just giving it to them.

*I wish that they would have involved us more in the process… A bit more support, not like the counselor, but so that you felt that the doctors were more available for questions, that would have been a great help (Child 7).*

Most of the children described that heredity had been discussed with the parents after the heart transplantation. In some cases, this had resulted in a genetic investigation, which was sometimes initiated not only by the family due to concerns about the future, but also by health care professionals. Some of the children did not want to know about the risk of heredity and refrained from further discussions about it.

### 4.2. The Family and Friends' Approaches

The children received most information from their parents, which pleased them. The parents left the brochures they had been given for the children to read. Few of the children used the Internet to find out more. A reason for not using the Internet was that they did not find specific information or that they did not want to find out more about heart transplantation.

During periods when the ill parent was hospitalized, the healthy parent was also away a lot. This meant that the siblings had to support each other. The children described how they tried to take each day as it came and solved the various problems at hand. If adult support was lacking, older siblings had to support the younger siblings. In retrospect, it was found that the younger siblings were given too much freedom for their own good. This occurred when the healthy parent spent time in the hospital with the sick parent or if the healthy parent also became ill. The older children could experience it as hard to go from being a young adult to being the parents' assistant in the family. Responsibilities such as washing, cleaning, shopping, cooking, dropping off and picking up siblings at kindergarten, homework, and school meetings were taken over by the older siblings in the family. The children described that the family needed support from society in order to relieve their burden.

*You could say that our roles changed, that I became my dad's right hand from having been a child. I mean from being his child to being his right hand (Child 1).*

Many children described how they continued with the same hobbies as they did before the parent became ill. Contact with friends and sports teams was an important support during this time. Being away from the family and forgetting about living with a sick parent in the family were important. Having a parent who was susceptible to infection at home meant that they could not bring friends home as before. Older siblings and grandparents dedicated a lot of time to the children's activities, for instance, by taking them to different leisure time activities. Older siblings and grandparents meant a lot by activated siblings, for example, by taking them to different activities. However, it also appeared that some had to stop their hobbies because of a weakened financial status in the family or because they were required to help out at home.

Grandparents, family friends, friends' parents, and their own friends were described as important support, before, during, and after the heart transplantation. Just having someone who asked them how they were and were present gave them a feeling of support.

*When dad was away, when he was in the hospital and mom was with him, we slept at a friend's house. We spent a lot of time with that friend and her parents, we were very close, and my sister slept there too, and they also knew a lot about the disease (Child 4).*

The children experienced that how well-informed relatives and friends were depended largely on the parents' ability and willingness to involve them. Health care professionals were not involved in providing information to friends and relatives, and the children experienced that when difficult situations arose and friends and relatives were to support the children, they were uninformed or had misunderstood the information and then gave the children the wrong information.

*Many did not quite understand and drew their own conclusions. It could be those closest to us who drew the wrong conclusions and thought that everything would end really badly and started planning for other things [death], which we found out, and that was not optimal (Child 7).*

There were also children who described that close relatives or family friends withdrew when the parent became ill. They experienced that some of their own friends did not dare to contact them and withdrew instead. The family members had to help themselves and they talked a lot with each other. Sometimes, other adult patients in the ward and their families became new friends instead.

*You can feel that many [friends] were sort of afraid of phoning, and now, afterwards, I can see that it is better to phone one time too many than just disappear out into the fringes (Child 10).*

The children described feelings such as fear, anxiety, confusion, relief, and joy in association with the heart transplantation. The children had detailed memories in connection with the heart transplant. They described what was happening and the physical location they were in during the hours when the parent received notice that they would have the transplant and until it was complete. Some children described being tired of once again not knowing the results or being able to relax. The fear of never seeing the sick parent again was imminent. The family support they received was important during those hours.

*I know that I felt really torn about having to wait for someone else to die so that my mom can live, and then it will be really hard for another family, just so that we can be really happy. I felt that that was not fair, but I still could not help wishing that my mom would live (Child 12).*

### 4.3. Society Approaches

The children described that the few times they experienced organized community support were when specific problems arose, and then only they themselves had received help, not the whole family. Parents and children were responsible for informing schools, other agencies, and workplaces. The children described that there was lack of professional support of the family when the children needed to contact the school, the social insurance office, and the authorities because of the ill parent. In general, no one talked about the children's situation, but sometimes a teacher would ask how they were. Some children described how one teacher became involved with them, and they described that this was more meaningful than going to the social worker.

*Of course it had an effect, it was not possible to focus as much on school work… and all that, because there was a constant worry about what would happen. It was tough, sure it was (Child 3).*

The children who were young adults and had started to work described the support from managers and colleagues as important. It was important that colleagues were aware of the situation and that they understood that their thoughts could sometimes be elsewhere.

Employers' understanding of the need to leave in a hurry without explanation was also important.

*Primarily, it was my work place that was my ventilation… (Child 1).*

When looking back, the children described that the family's finances had probably been affected by loss of income due to the parent's illness. They perceived that eating in restaurants and food shopping occurred less frequently, and it was not possible to buy expensive gifts, mobile phones, or new computers. None of the children's parents spoke openly with them about the deteriorating finances, but they understood anyway. Later, they heard of counselors who had helped families to apply for funds, housewives who had to go back to work, and some who had gone on sick leave to be with the sick parent in the hospital. Someone also described how they had to use their salary to contribute to the household when they started working but that they did not connect this to the parent's illness at the time.

There were also older children who described that they had to contact the insurance office to ensure that the family received money, as the parent was too ill to manage this.

Professional support was missing in these situations. The children described that when they needed to contact the social security office and authorities because of the sick parent, there was lack of professional support of the family.

*It could have been different if I had received support, counseling. It was a whole new world to me, for instance, the social security office, which I had never been in contact with before, and I did not know how to best deal with all the paperwork. I could also have received some help with a crisis, because many years later I experienced a crisis (Child 12).*

## 5. Discussion

### 5.1. Result Discussion

The results indicate that the family around the child was unsteady. There was lack of information and support to children with a heart transplant parent. Information to the children's schools was usually provided by the child or the parent. The social environment included grandparents, friends, and sports clubs that offered great support. The children took a lot of responsibility at home, particularly the older siblings. The children experienced great fear in association with the surgery and relief and joy when it was over. Our results show that health care organizations did not provide children of transplanted patients with information or support and that these were in most cases provided by the parents. This finding was confirmed by both transplant patients and other adults, who described that there was particular lack of tailored information and support to children and adolescents in the context of parent transplant [5, 7, 24]. Earlier studies have shown that health care professionals should communicate with patients and children about diagnosis, the short- and long-term implications of the disease, and the children's needs [25, 26].

The child or the parent usually informed the school, and they normally initiated contact without any help from the health care professionals. At school, the children sometimes felt that they were different because of their situation, and they had to manage their homework on their own. Some teachers, but far from all, asked how they felt. A study about young caregivers showed that incomprehension can cause gossip and rumors among peers at school, as well as among others [27]. In order to support the child, the environment needs to be sensitive to what the child wants and can handle and be open about their feelings. The social environment generally included grandparents, friends, and sports clubs that offered great support. Young people wanted to get in touch in real life, and they sometimes also needed health care or social care for direct help. Although web-based support cannot deal with such needs, it can help to fulfill some of them. Web-based support may be a suitable way to meet the need for knowledge and fulfill some of the needs for communication [11]. Further, digital forums could be used to expand the children's social network and also help them to get in touch with others in a similar situation.

The children worked hard to adapt to the stressful life situation imposed on them by having a severely ill parent and to preserve and care for the family system. The children took great responsibility at home, particularly the older siblings. They had to take responsibility for the younger siblings, the household, and even the family finances as they had to contact the social insurance agency. The grandparents often visited and helped the family with the household. Previous research has found that young carers view the experience of caring for a parent as positive overall but identified opportunities where professionals could assist them in overcoming the stigma and challenge of balancing childhood and adolescent development within this context [27].

In some families, finances were affected and the children were not able to have the most recent technology or take-away food. Holidays were also affected. Studies have confirmed that patients and their partners have financial problems in connection with the heart transplantation [6, 28]. Although most of the children seemed to manage rather well, they were all strongly affected by the disease [14].

All children expressed similar and complementing views about uncertainty and anxiety, separation, disruption of family life, their desire for normality, and the importance of social support. Differences were evident in the way that some children of heart transplant patients managed by adapting to the changing situations, whereas others tended to avoid them [26]. Parents with cancer have indicated that they wanted support from the health care professionals on how to manage changes in their child's behavior [29]. This support should also apply to transplant care.

The children experienced great fear in association with the surgery and relief and joy when it was over. Parental transplant status had positive and negative effects. No evidence was found regarding signs or symptoms of depression. The children of transplant candidates worried about “something going wrong” [30]. They received adequate information regarding heredity. Some wanted to know but not all. It is of great importance to develop psychosocial support for families with a seriously ill parent, focusing on facilitating communication and empowering parental function, and help the children to share their concerns and thoughts. In a previous study, children, parents, and health professionals reported that interventions had several positive impacts on children's and parents' psychosocial well-being [31]. Work is underway to transfer well-established family intervention programs from psychiatric to somatic care [32], the need for which is confirmed by this study.

It is of great clinical importance to develop psychosocial support for families with a seriously ill parent focusing on facilitating communication and empowering parental function and helping the children to share their concerns and thoughts and to involve children in a structured and deliberate manner. Using this result as a starting point, primary health care, municipalities, bot social services and schools, local health care, and patient associations can develop support programs (e.g., via Internet) focusing on patients and their families, particularly the children.

### 5.2. Methodological Considerations

There are several limitations to this study that have to be acknowledged. First, eight transplant parents (patients) did not want their children to be interviewed. According to one parent, the reason was that he did not want to stir up those emotions again. All patients who had had heart transplants in one of the two centers in Sweden and who had children were asked to participate. This improved transferability to other children in the same situation. To collect data, telephone interviews were used as a way to bridge the geographical distance between the participants and interviewers. In addition, this strategy allowed the interviews to be done independently of the participants' location and provided them with greater anonymity. However, not being able to hold face-to- face interviews could have affected the results as the rich data generated in the interaction between the participant and the interviewer was missing [21]. During the analysis, all researchers read the material several times to ensure that the meaning units and codes were consistent with the results. The material was also criticized so that improvements could be made throughout the process, which increased credibility [23]. In future research, other interview methods may provide more knowledge about this population, especially if the interviews are made close in time with the parent's heart transplant. However, the interviewer must have good knowledge of children and adolescents and be aware of the difficulties of interviewing a young population [33].

## 6. Conclusion and Clinical Implications

To our knowledge this is the first study to address this topic. Our results show that children with a heart transplant parent need more support. The roles in the family are changing and the children need to understand the situation to prevent anxiety, depression, and somatic complications. Today, research regarding how support can be given to these children is missing and the role of the multidisciplinary team is unknown. The multidisciplinary team should give more attention to the partner and children involved in the organ transplantation process, due to the emotional and social burden.
